# Intra and latero-sellar carotid aneurysm mimicking a pituitary adenoma

**DOI:** 10.11604/pamj.2015.22.150.8043

**Published:** 2015-10-16

**Authors:** Naima Bouznad, Nawal El Ansari

**Affiliations:** 1 Department of Endocrinology, Diabetology and Metabolic Diseases, PCIM Laboratory, Faculty of Medicine and Pharmacy Marrakech, University Hospital Center of Mohamed VI, Marrakech, Morocco

**Keywords:** Carotid aneurysm, pituitary adenoma, hyperprolactinemia

## Image in medicine

The aneurysm is a dilation of an artery focused secondary to a parietal structural modification. The intracranial carotid aneurysm is a rare disease, which exposes to serious risks, we report a case. A 46-year-old women, followed for infertility last 5 years, which presents a secondary amenorrhea 6 months ago, without functional signs of anterior pituitary deficiency or signs of intracranial hypertension or galactorrhea. hormonal analysis shows hyperprolactinemia to 97 ng/ml. Hypothalamic-pituitary magnetic resonance imaging (MRI) shows a round formation, intra and latero sellar right hypointense in T1 and T2 (17 * 12mm) with contrast enhancement after injection (A), it is in intimate contact with right carotid cavernous and lifting the head of a right optic chiasm and compression at the bottom of the cavernous sinus (B). The magnetic resonance angiography is in favor of an unruptured aneurysm carotid (C, D). The carotid aneurysm is a diagnosis that is rare, usually asymptomatic and discovered during a major complication (break), it should be mentioned in the evocative radiological appearance. Screening can be organized and magnetic resonance angiography is therfore the first line examination. The arteriography is the key examination that allows the diagnosis and endovascular treatment. Good technical knowledge of these imaging methods, artifacts and pitfalls images allow reliable detection of intracranial aneurysms and performing an accurate account to be provided to clinicians. We insist, through this observation, on the interest not to disregard this diagnosis.

**Figure 1:**
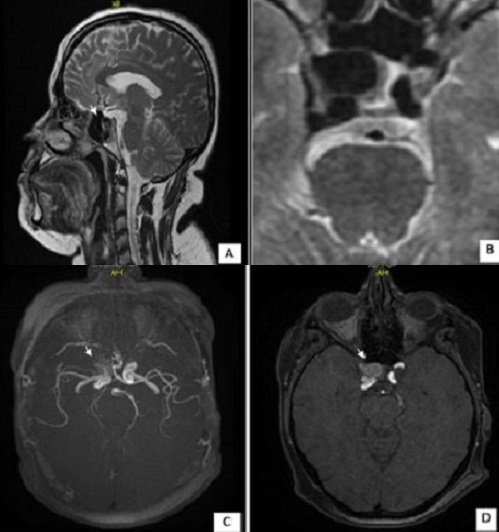
Intra and latero-sellar carotid aneurysm

